# Reverse innovation in diabetic foot disease prevention: Rethinking family and community roles

**DOI:** 10.1111/dme.70336

**Published:** 2026-04-18

**Authors:** Joseph Ngmensegre Suglo, kirsty Winkley, Doreen Asantewa Abeasi, Jackie Sturt

**Affiliations:** ^1^ Better Health & Care hub King's College London London UK; ^2^ Florence Nightingale Faculty of Nursing, Midwifery and Palliative Care King's College London London UK; ^3^ Department of Nursing and Midwifery Presbyterian University Agogo Ghana

**Keywords:** diabetes, diabetic foot ulcers, family caregivers, foot care, Ghana, task sharing

Diabetic foot disease persists as a major global complication of diabetes, requiring approaches that move beyond conventional clinic‐centred prevention.^1^ Our feasibility trial in Ghana demonstrated that family‐centred, context‐appropriate foot‐care education delivered by nurse‐educators can strengthen preventive behaviours and self‐efficacy meaningfully.^2^, ^3^ The Ghanaian family centred foot‐care model, developed in the context of limited specialist availability and significant literacy variation, offers a compelling example of reverse innovation, insights generated in a low‐resource setting that carry significant relevance for high‐income health systems now facing their own pressures. Without restating the findings of the Ghanaian feasibility trial, which are reported in detail elsewhere,^2^ we rather use the results to facilitate reflection on how preventive foot care responsibility is distributed within health systems, how household and community actors are positioned relative to formal services, and how lessons from resource‐constrained settings might inform debates about sustainability, workforce capacity and equity in high‐income contexts. The commentary uses early insights to challenge assumptions about the location and participants of diabetic foot disease prevention, not to claim immediate generalisability.

## 1 A RESOURCE‐CONSCIOUS TASK‐SHARING MODEL FOR PREVENTIVE DIABETIC FOOT CARE

The intervention in Ghana was co‐designed with people living with diabetes, their caregivers and nurses, and delivered using simple visual materials, practical demonstrations and a strong emphasis on household‐level responsibility for prevention. In our trial, patient–caregiver dyads engaged actively with daily foot inspection, basic foot and nail care and home‐based screening for loss of protective sensation. Efficacy signals at 12 weeks suggested improvements in foot‐care behaviour, self‐care efficacy, diabetes knowledge and caregiver distress, an encouraging set of early markers for a model that requires comparatively few resources to implement.^2^ This approach resonates with well‐established principles of patient‐ and family‐centred care models within chronic disease care, including diabetes management.^3^, ^4^, ^5^, ^6^, ^7^ The Ghanaian model differs because rather than focusing primarily on shared decision‐making within clinical encounters, it deliberately operationalises family involvement in routine preventive tasks, including daily foot inspection and basic sensory screening supported by structured training, visual tools and clear escalation thresholds. This moves beyond relational partnership to a clearly defined redistribution of preventive work, raising new questions about support and accountability that requires specific consideration.

Conversely, any proposal to extend preventive care into households must engage seriously with equity considerations. There is emerging evidence suggesting how task‐shifting and home‐based care models can inadvertently transfer responsibility from formal health systems to unpaid caregivers, who are disproportionately women and individuals already experiencing social disadvantage.^8^ Without adequate support, such models risk worsening existing inequities rather than alleviating them. Moreover, not all individuals with diabetes live in family settings or have access to reliable informal support. For those who experience social isolation or face barriers to accessing responsive clinical services, household‐based monitoring may offer limited benefit unless accompanied by parallel investments in access, continuity and specialist capacity. Home‐level surveillance using the Ghanaian approach may only improve outcomes if it is embedded within a health system that responds promptly when foot problems are identified.

Although designed for a context where podiatrists and diabetes educators are scarce, the logic of the Ghanaian approach may be applicable across diverse health systems. High‐income countries increasingly challenge with podiatry capacity constraints, growing case complexity and substantial inequalities in health literacy, factors that collectively limit the reach of traditional clinic‐based foot surveillance.^9^ The simplicity of the Ghanaian model, and its grounding in everyday life, addresses these challenges directly. Rather than orienting prevention around specialist encounters, it equips people with diabetes and their families to monitor and respond to early warning signs long before they reach the threshold of ulceration or infection. In this respect, the intervention reframes prevention as something generated within households and supported by professionals, rather than owned by professionals and occasionally extended to households. There is also a clear alignment between this model and evolving priorities in high‐income settings. For instance, the ‘Fit for the Future: 10 Year Health Plan for England’ aims to make the NHS more sustainable and patient‐centred through moving care from hospitals to communities and focusing on prevention rather than just reactive treatment.^10^ Global non‐communicable diseases (NCDs) strategies increasingly emphasise community‐based prevention, earlier detection and redistribution of tasks across the workforce. In Ghana, national efforts supported by WHO have focused on primary‐care training, routine screening and strengthening integrated NCD services^11^; these priorities mirror the shifts in high‐income systems that seek to relieve pressure on specialist services while building capacity closer to home.

## 2 GLOBAL LEARNING AND FUTURE DIRECTIONS

A strength of the Ghanaian intervention is the treatment of family caregivers not as auxiliary helpers but as partners whose involvement can reshape the trajectory of chronic illness management. The model is rooted in the cultural normalcy of family involvement in health, but the premise is broader: families are consistently present in the lives of people with diabetes, often more so than clinicians. Structured, intentional engagement of caregivers can enhance monitoring, adherence, shared understanding and confidence, outcomes that clinical consultations alone may be less able to achieve. At the same time, the transfer of such approaches across settings requires careful attention to context. Family structures, caregiving norms, professional boundaries and medicolegal frameworks may vary significantly between countries and health systems. Not all individuals with diabetes have access to willing or able family caregivers, and responsibility for preventive foot care cannot be assumed to rest solely within households. In such contexts, diabetic foot care roles may be played by community health workers, peer supporters or other trusted non‐specialist actors. The core transferable principle from the Ghanaian experience therefore is the deliberate extension of preventive foot care beyond the clinic through clearly governed, supported and accountable relationships. Importantly, family or community‐supported monitoring functions as an adjunct to, rather than a replacement for, specialist foot services.

Reverse innovation challenges hierarchical assumptions about where expertise originates. Calls to decolonise global health have highlighted how high‐income systems sometimes overlook knowledge emerging from low‐income contexts, despite the fact that such settings frequently pioneer robust, frugal and behaviourally attuned approaches to care.^12^ Innovations developed under constraint, as in Ghana, can reveal what is essential, scalable and resilient, qualities urgently needed in systems facing rising chronic disease burden, limited specialist availability and persistent inequities. Meaningful progress requires a willingness to learn from contexts historically marginalised in knowledge production.

Adopting such approaches does not imply replicating Ghana's model wholesale but rather drawing on its principles. Both low and high‐income countries can implement streamlined, nurse‐led education; integrate family members into preventive foot‐care pathways; use clear visual tools to support people with varying health‐literacy levels; and introduce simple home‐based screening to extend surveillance between clinic appointments. Incorporating families into routine diabetes self‐management support may be especially beneficial for older adults, people with multi‐morbidity, or those facing socioeconomic or linguistic barriers, groups at heightened risk of foot complications. The importance of early detection and home‐level vigilance cannot be overstated. In our Ghana study, caregivers identified previously unrecognised loss of protective foot sensation, prompting timely clinical review. This is precisely the type of early action that prevents ulceration and subsequent amputation, and it reflects the strength of a model that places monitoring closer to where risk accumulates.

Both low‐ and high‐income settings could pilot this family involvement approach through existing primary‐care, Quality and Outcomes Framework (QOF) processes, community nursing, or integrated diabetes care pathways with clear fast track referral pathways for specialist escalation. Evaluation should focus not only on behavioural outcomes but also on acceptability, clinician workload and equity of access. Comparative implementation studies between Ghana and other high‐income settings could explore how cultural, organisational and policy contexts shape the success of caregiver‐led diabetic foot ulcer prevention models. Such future studies should also integrate health‐economic analyses alongside clinical and behavioural outcomes to assess the economic appeal of this model of care. While the Ghanaian intervention was designed to fit into a resource limited context, using brief nurse‐led group sessions and low‐cost logistics, formal economic evaluation is required to assess its potential cost savings from preventable foot ulcers and amputations, and the distribution of costs between health systems and households.

## 3 CONCLUSION

The broader lesson is that successful prevention of diabetic foot problems may be improved when health systems recognise the family environments in which chronic illness is lived. Foot‐care cannot depend solely on episodic contact with specialists when the most decisive moments occur in the home environment. High‐income countries grappling with rising risk, service pressures and persistent inequalities should see the Ghanaian model not as born out of scarcity but as a blueprint for sustainable, human‐centred prevention. The model has shown that families, when supported appropriately, can extend the reach of the health system.

## FUNDING INFORMATION

No funding was received for the preparation of this manuscript.

## CONFLICT OF INTEREST STATEMENT

The authors declare no conflicts of interest.

## Data Availability

Data sharing not applicable to this article as no datasets were generated or analysed during the current study.
